# Genome-Wide Analysis of Serial Passage of the Infectious Bronchitis Virus Reveals Evolutionary Dynamics Underlying Attenuation and Immunogenicity

**DOI:** 10.3390/vaccines14060467

**Published:** 2026-05-24

**Authors:** Joaquín Williman, Gonzalo Tomas, Ariel Vagnozzi, Claudia Techera, Sebastián Brambillasca, Ruben Pérez, Ana Marandino

**Affiliations:** 1 Sección Genética Evolutiva, Departamento de Biología Animal, Instituto de Biología, Facultad de Ciencias, Universidad de la República, Iguá 4225, Montevideo 11400, Uruguay; jwilliman@fcien.edu.uy (J.W.); gtomas@fcien.edu.uy (G.T.); ctechera@fcien.edu.uy (C.T.); 2 Instituto de Virología e Innovaciones Tecnológicas, Centro de Investigaciones en Ciencias Veterinarias y Agronómicas (CICVyA), Instituto Nacional de Tecnología Agropecuaria-Consejo Nacional de Investigaciones Técnicas (INTA-CONICET), Hurlingham 1686, Buenos Aires, Argentina; vagnozzi.ariel@inta.gob.ar; 3 Unidad Académica de Avicultura, Departamento de Producción Animal y Salud de los Sistemas Productivos, Facultad de Veterinaria, Universidad de la República, Ruta 8 y Ruta 102, Montevideo 13000, Uruguay; sebastian.brambillasca@fvet.edu.uy

**Keywords:** IBV, serial passage, attenuation, Spike protein, viral evolution, quasispecies, genomic changes, vaccine development

## Abstract

**Background/Objectives**: Serial passage in embryonated eggs is widely used to attenuate the infectious bronchitis virus (IBV) for vaccine production; however, the evolutionary processes underlying attenuation and immunogenicity remain incompletely understood. Here, we analyzed genome-wide viral evolution during serial passages to investigate how mutations emerge, persist, are lost, or become fixed over time and how these dynamics relate to changes in pathogenicity and immunogenicity. **Methods**: Deep sequencing was performed on 11 representative serial passages (P2–P79) of the UY/11/CA/18 strain, including two derivative lineages: P7 VIR (virulent) and P53 VAC (attenuated and immunogenic). **Results**: This study identified an early adaptive phase characterized by a limited set of mutations potentially associated with genome replication, viral RNA processing, and virion assembly, including a key change in non-structural protein 14 and variants in M and 3c (E). This phase was followed by a broader expansion of the variant spectrum across replicase genes and delayed accumulation of Spike protein variants. Most Spike changes emerged during later passages and exhibited transient dynamics, and only a subset reached a high frequency after the establishment of early replicase- and structural-associated changes. Consistent with these dynamics, P7 VIR diverged before the late accumulation of Spike variants and retained a pathogenic phenotype, whereas P53 VAC diverged after the emergence of early high-frequency variants but before the extensive late-stage Spike variation observed in P79, which was associated with reduced immunogenicity. **Conclusions**: These findings support a multi-step model of IBV attenuation in which progressive filtering of genome-wide variation shapes distinct evolutionary outcomes during serial passages. This evolutionary framework provides insight into the relationship between attenuation and immunogenicity and may help guide the rational design of live attenuated vaccines.

## 1. Introduction

Infectious bronchitis virus (IBV), a member of the genus *Gammacoronavirus*, is among the most economically significant pathogens affecting the global poultry industry. Since its first description nearly 90 years ago [1,2], IBV has remained a major challenge due to its high genetic variability and rapid evolution. The virus has a single-stranded, positive-sense RNA genome (~27.6 kb) with high mutation and recombination rates, driving the continuous emergence of new variants and genotypes [3,4,5].

IBV control relies primarily on vaccination, particularly with live attenuated vaccines produced by serial passage in embryonated chicken eggs. This process promotes adaptation to the embryonic environment, typically resulting in reduced pathogenicity in chickens while preserving replication and immunogenicity [6,7]. However, attenuation remains an empirical and only partially predictable process. Insufficient passaging may leave residual pathogenicity, whereas excessive passaging can lead to over-attenuation and loss of immunogenicity. Understanding the genetic and evolutionary mechanisms that govern this balance is therefore critical for improving vaccine design and performance.

Accumulating evidence indicates that attenuation in IBV is a multigenic, strain-specific process. Although the Spike (S) protein, particularly its S1 subunit, plays a central role in antigenicity and host interaction [8], it is not solely responsible for virulence. Experimental studies have demonstrated that replicase genes, particularly within ORF1a/1ab, can determine pathogenicity independently of the S gene [9,10,11]. Additional contributions from structural and accessory proteins have also been reported, including nucleotide substitutions and deletions affecting 3a, 3b, and 5a/5b ORFs [12,13]. Together, these findings support a model in which attenuation results from interactions among mutations across the genome, rather than from the independent effects of individual mutations.

Beyond consensus-level changes, IBV populations are organized as quasispecies, defined as dynamic distributions of closely related variants generated by error-prone RNA replication, whose composition is shaped by the interplay between selective pressure and stochastic processes such as genetic drift. Studies on ArkDPI-derived vaccines have demonstrated rapid in vivo selection of subpopulations [14,15], and adaptation to alternative substrates can reduce diversity and promote the fixation of specific variants [16]. These observations suggest that attenuation is likely influenced not only by the presence of specific mutations but also by the temporal dynamics governing variant emergence, competition, and fixation.

Despite these advances, the evolutionary processes underlying IBV attenuation remain incompletely understood. In particular, how mutations emerge, fluctuate, are retained or lost, and eventually become fixed during serial passage, and how these dynamics relate to changes in pathogenicity and immunogenicity, have not been systematically characterized at a genome-wide, time-resolved scale.

In this study, we analyze the genomic evolution of an IBV strain from genotype GI-11, a lineage of major epidemiological relevance in South America. Using next-generation sequencing (NGS), we combine consensus genome analysis with tracking of minor variant allele frequencies across serial passages in embryonated eggs. This approach enables a detailed, time-resolved characterization of viral population dynamics. We also performed in vivo evaluation of the pathogenicity and immunogenicity of selected passages infecting SPF birds. This allows us to investigate how genome-wide mutation patterns are associated with attenuation and immunogenicity during serial passage.

## 2. Materials and Methods

### 2.1. Virus and Serial Passaging in Embryonated Chicken Eggs

The Uruguayan IBV strain UY/11/CA/18 (GenBank accession no. MF421320.1; lineage GI-11) was serially passaged 79 times in 10–11-day-old specific-pathogen-free (SPF) embryonated chicken eggs. At each passage, three eggs were inoculated via the allantoic cavity with 0.2 mL of a 1:10 dilution of allantoic fluid in phosphate-buffered saline (PBS) supplemented with penicillin (100 IU/mL) and streptomycin (100 μg/mL), then incubated at 37 °C for 72–96 h. Allantoic fluid from each passage served as the inoculum for the next. Every ten passages, embryo mortality and IBV-associated lesions were evaluated, and viral replication was confirmed by RT-qPCR.

### 2.2. Virus Titration in Embryonated Chicken Eggs

**Figure 1:**
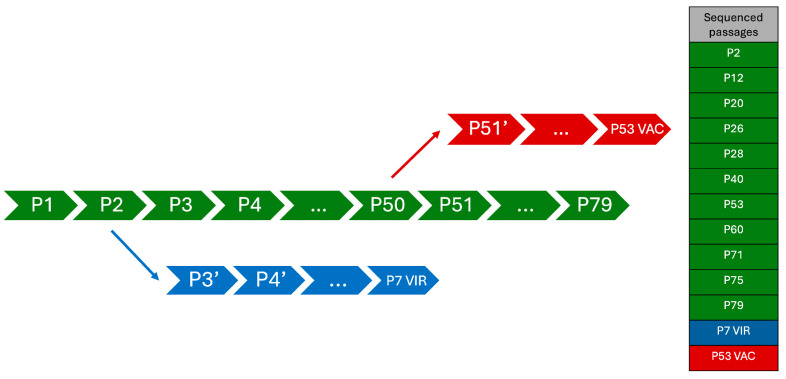
Serial passage scheme of IBV strain UY/11/CA/18. Schematic of serial passage of IBV strain UY/11/CA/18 in embryonated chicken eggs (P2–P79). Sampling points for genomic analysis are indicated. Two derivative lineages originating from earlier-divergence points were defined: P7 VIR (blue), representing an early, virulent branch, and P53 VAC (red), corresponding to an attenuated, immunogenic lineage. Both lineages were generated for virus titration in chickens. The diagram illustrates the temporal progression of passages and the divergence of phenotypically distinct viral populations.

To obtain passages with clear embryo lesions suitable for titration, additional serial passages were performed starting from P2 and P50. After phenotypic characterization, the resulting passages used for titration and subsequent animal infections were retrospectively labeled P7 VIR and P53 VAC for clarity (Figure 1). The infectious titers of P7 VIR, P53 VAC, and P79 were determined in 11-day-old SPF embryonated eggs. Tenfold serial dilutions (10^−3^ to 10^−10^) were inoculated into the allantoic cavity (0.2 mL per egg; *n* = 5 eggs per dilution) and incubated at 37 °C for 7 days. Embryos that died within 24 h post-inoculation were excluded from the analysis. The 50% embryo infectious dose (EID_50_) was calculated using the Reed–Muench method [17].

### 2.3. Pathogenicity Evaluation in SPF Chickens

Three independent experiments were conducted, each using forty SPF chicks at one day of age (*n* = 40 per experiment; total *n* = 120). Chicks were randomly assigned in equal numbers to two groups: an experimental group (P7 VIR, P53 VAC, or P79, depending on the experiment) and a non-infected control group. Birds were housed in negative-pressure isolators with HEPA-filtered air at 37 °C, with ad libitum access to feed and water.

Birds were inoculated with a total dose of 2 × 10^4^ EID_50_, administered via the intraocular (both eyes) and intranasal (both nostrils) routes (50 µL per route). Non-infected birds received PBS.

Clinical signs were monitored daily for 21 days. At 2, 7, 14, and 21 days post-infection (dpi), 5 birds per group were euthanized by cervical dislocation, and samples from the trachea and cecal tonsils were collected to quantify viral load.

### 2.4. Serology

At 7, 14, and 21 dpi, blood samples were collected before euthanasia via the occipital sinus (7 dpi) or by cardiac puncture (14 and 21 dpi). IBV-specific serum antibodies were measured using a commercial ELISA kit (IDEXX IBV Ab Test; IDEXX Laboratories, Westbrook, ME, USA) according to the manufacturer’s instructions. Samples were considered positive based on the manufacturer’s recommended cutoff criteria.

### 2.5. Viral Load Quantification by RT-qPCR

Tissues (trachea and cecal tonsils) were weighed and homogenized in PBS with zirconium beads using a TissueLyser LT (Qiagen, Hilden, Germany). RNA was extracted using the QIAamp Viral RNA Kit on a QIAcube system (Qiagen).

Absolute quantification was performed using the ZymoScript One-Step RT-qPCR Kit (Zymo Research, Irvine, CA, USA) targeting the IBV 5′ UTR [18]. Standard curves were generated from in vitro-transcribed RNA spanning 10^2^–10^8^ copies/µL. Reactions were run in duplicate on a Mic qPCR system (BioMolecular Systems, Upper Coomera, QLD, Australia). Each run included negative controls (no-template and extraction controls).

Viral loads were reported as genome copies per gram of tissue. Technical duplicates were averaged to obtain a single value per sample. Samples below the detection limit (<100 copies/g) were assigned 50 copies/g for statistical analysis. Data were log_10_-transformed before analysis.

Residual normality and homogeneity of variance were assessed with the Shapiro–Wilk and Levene tests, respectively. For tracheal samples, viral loads were analyzed using a two-way ANOVA with viral passage (P7 VIR vs. P53 VAC) and dpi as fixed factors, with their interaction included. For cecal tonsils, comparisons at each time point were performed with the Mann–Whitney test. Because P7 VIR and P53 VAC were evaluated in independent experiments, statistical comparisons were interpreted considering that all experiments were conducted under standardized conditions, including animal source and age, housing conditions, inoculation procedures, sampling schedule, and analytical workflow. All analyses were conducted in R (version 4.4.2), with significance set at *p* < 0.05.

### 2.6. Passage Sequencing and Bioinformatics Analysis

Viral RNA from IBV strain UY/11/CA/18, corresponding to selected passages between P2 and P79 (Figure 1), including the derivative lineages P7 VIR and P53 VAC, was subjected to next-generation sequencing (NGS).

Reverse transcription and double-stranded synthesis were carried out using the Maxima H Minus Double-Stranded cDNA Synthesis kit (Thermo Fisher Scientific, Waltham, MA, USA) and 13 µL of extracted RNA.

Sequencing libraries were prepared using the Nextera DNA Flex Library Prep Kit (Illumina, San Diego, CA, USA), and paired-end sequencing was performed on an Illumina MiniSeq platform, targeting a minimum coverage depth of 949×.

Raw reads were quality-filtered using BBduk, Version 38.84, and mapped to the reference genome (GenBank accession no. MF421320.1) using Minimap2 [19]. Variant calling was performed in Geneious Prime v2026.0.2 [20] and manually inspected.

Variant allele frequencies (VAFs) were calculated for each genomic position. Variants were retained based on minimum coverage ≥ 100× and frequency ≥ 5% to minimize the impact of sequencing errors.

Mutation tables were curated and normalized using custom Python 3.12.1 scripts. VAF trajectories across passages were analyzed and visualized using pivot tables, heatmaps, and bubble plots, with variants categorized into frequency classes (5–50%, 50–85%, and ≥85%).

## 3. Results

### 3.1. Evaluation of the Attenuation Level of the GI-11 Uruguayan Strain

To evaluate attenuation during serial egg passage, SPF chickens were inoculated with passages representing different stages of adaptation: P7 VIR (early-stage, virulent lineage), P53 VAC (intermediate-stage, attenuated and immunogenic lineage), and P79 (late-stage passage associated with reduced immunogenicity).

#### 3.1.1. Clinical Signs

Birds infected with P7 VIR developed mild respiratory signs, characterized by tracheal rales observed at 7 dpi in all individuals examined, which resolved within 2–3 days. No additional clinical signs were observed. Birds inoculated with P53 VAC or P79 remained clinically normal throughout the experiment, as did those in the non-infected control group.

#### 3.1.2. Serological Response

All birds infected with P7 VIR developed detectable IBV-specific antibodies by 21 dpi (5/5). Seroconversion was already evident at 14 dpi in a subset of animals (3/5).

The P53 VAC group showed a delayed and reduced serological response, with antibodies detected in 1/5 birds at 14 dpi and 4/5 birds at 21 dpi.

No IBV-specific antibodies were detected at any time point in birds inoculated with P79. All control animals remained seronegative.

#### 3.1.3. Viral Replication Dynamics

**Figure 2:**
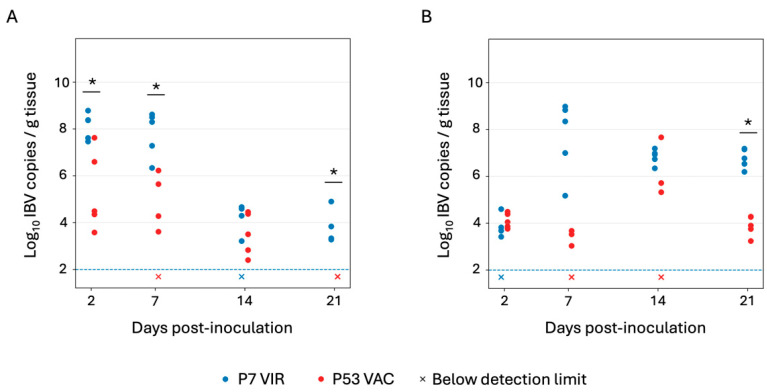
Viral load dynamics in virulent and attenuated IBV passages. Viral loads in the trachea (**A**) and cecal tonsils (**B**) of one-day-old SPF chickens inoculated with IBV passages P7 VIR (virulent, blue) and P53 VAC (attenuated and immunogenic, red) were measured from 2 to 21 days post-infection. Viral genome copies were quantified by RT-qPCR at multiple time points. Distribution plots include values below the detection limit (shown as crosses), and the detection threshold is indicated. Asterisks indicate statistically significant differences between P7 VIR and P53 VAC at each time point (*p* < 0.05); the dotted line indicates the detection threshold.

Viral load dynamics differed across tissues and inocula. In tracheal samples, two-way ANOVA revealed significant effects of inoculum, dpi, and their interaction (*p* < 0.05), indicating that viral replication dynamics differed between groups over time. Post hoc comparisons showed significantly higher viral loads in P7 VIR at 2, 7, and 21 dpi, whereas no significant differences were observed at 14 dpi (Figure 2A).

In cecal tonsils, viral loads were generally lower. Nonparametric comparisons showed significantly higher viral loads in P7 VIR at 21 dpi, whereas differences at earlier time points were not statistically significant (Figure 2B).

### 3.2. Genome-Wide Variants Detected During Serial Passages

A total of 175 unique genomic variants were identified across all analyzed passages (Table S1). Of these, 33 variants reached frequencies above 50% in at least one passage, and 21 exceeded 85%, consistent with near fixation.

At the genomic level, replicase genes accounted for the highest number of variants (*n* = 102), primarily at low to intermediate frequencies. Structural genes accumulated 56 variants, with the S gene showing the highest count (*n* = 28). To account for differences in gene length, variant counts were normalized as variants per kilobase (variants/kb). This analysis revealed that although the S gene accumulated the largest absolute number of variants, some shorter accessory ORFs exhibited proportionally higher mutational densities. After normalization, the highest mutational densities were observed in nsp11-coding sequences and ORFs 3c, 4c, 4b, and 6b. Overall, these findings indicate that genomic variation was unevenly distributed across the viral genome during serial passage.

**Figure 3:**
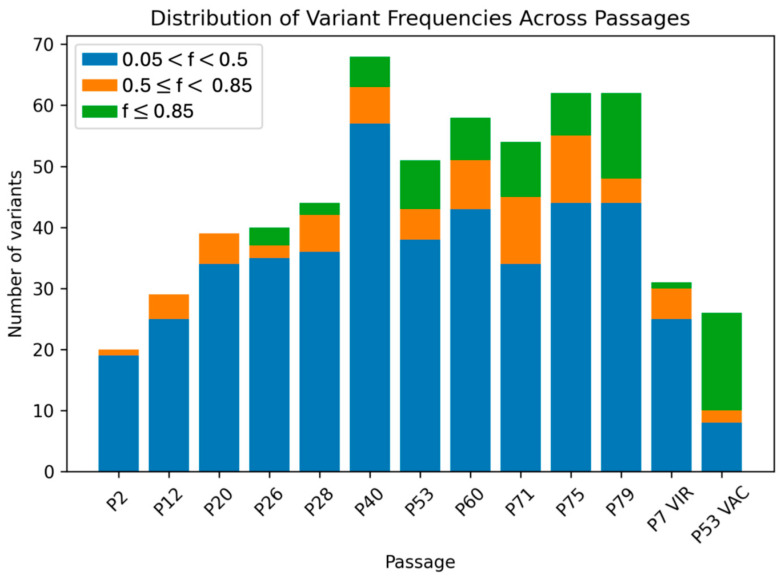
Distribution of variant allele frequencies across passages. Stacked bar plot showing the distribution of genomic variants across serial passages by variant allele frequency (VAF) category: low-frequency (0.05 < VAF < 0.5), intermediate-frequency (0.5 ≤ VAF < 0.85), and high-frequency or near-fixed variants (VAF ≥ 0.85). Passages are ordered chronologically. Derivative lineages (P7 VIR and P53 VAC) are included for comparison.

Most variants (*n* = 142) remained below 50% frequency throughout the experiment. Analysis of variant frequency distribution across passages revealed a progressive transition from predominantly low-frequency variants in early passages to the emergence of intermediate- and high-frequency variants in later stages (Figure 3).

High-frequency variants were most prominently represented in the latest passage (P79) and in the P53 VAC lineage, whereas P7 VIR exhibited a profile more consistent with early passages.

**Figure 4:**
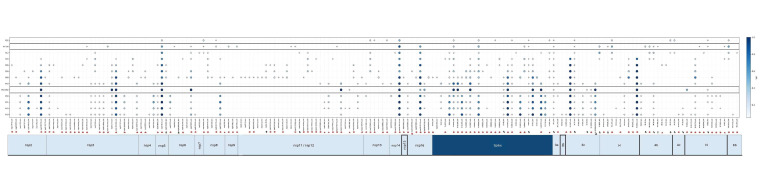
Genome-wide bubble heatmap of variant dynamics. Genome-wide bubble heatmap of variant allele frequencies (VAFs) across serial passages. Rows represent passages in chronological order, and columns represent variants organized by coding sequence (CDS) and genomic position. Bubble size and color intensity are proportional to VAF. Variants with VAF < 5% are not shown. Symbols below labels indicate predicted effects: red circles, non-synonymous SNPs; triangles, deletions; arrows, insertions; zigzags, frameshifts; and red asterisks, premature stop codons. Synonymous substitutions are indicated only by labels. Geneious Prime annotations: “non”, synonymous; “sub”, amino acid substitution; “tru”, truncation; “fra”, frameshift. The plot shows the distribution and frequency dynamics of variants during serial passage.

Most variants (*n* = 149) corresponded to single-nucleotide substitutions (SNPs), including 107 non-synonymous and 42 synonymous changes. Synonymous nucleotide substitutions were broadly distributed and generally remained at low frequencies. In contrast, non-synonymous substitutions were more frequently represented among intermediate- and high-frequency variants, particularly in the S gene and selected replicase genes (Figure 4).

Insertions and deletions (indels) were rare, mostly short (1–3 nucleotides), and remaining at low frequencies. A notable exception was a 21-nucleotide in-frame deletion in the 3c (E) gene that increased in frequency across passages (Table S1).

A nonsense mutation in the S gene, caused by a G→T substitution introducing a premature stop codon that truncates the protein (S-1161_tru), was detected at low frequencies (0.11–0.16) between P2 and P40 but was not observed in later passages or in the derivative lineages.

### 3.3. Dynamics of Variant Frequencies During Serial Passage

Serial passage resulted in a consistent temporal pattern of variant emergence and frequency change, broadly organized into three sequential phases (Figure 3 and Figure 4).

In the early phase (P2–P20), viral populations were dominated by low-frequency variants (VAF 0.05–0.5), with only 5 variants reaching intermediate frequencies (VAF ≥ 0.5). These variants were limited to a few loci, primarily in replicase genes (notably nsp14) and structural genes such as M and 3c (E). No variants reached the high-frequency category (VAF ≥ 0.85) during this stage.

During the intermediate phase (P20–P53), the number of detectable variants increased, including variants reaching intermediate and high frequencies. This phase was characterized by the coexistence of multiple variants within the viral population and the emergence of additional non-synonymous mutations across several replicase genes, including nsp3, nsp5, nsp14, and nsp16.

In the late phase (P53–P79), the number of detectable variants decreased, largely due to the loss of low-frequency variants, while previously established variants generally persisted or reached fixation. Notably, this phase showed an increased representation of variants in the S gene, several of which rose in frequency after P60, indicating a temporal shift in the genomic regions most affected during serial passage.

### 3.4. Divergence of Virulent and Attenuated Lineages

Incorporation of P7 VIR and P53 VAC into the temporal framework revealed distinct patterns of variant composition and frequency dynamics (Figure 4).

P7 VIR exhibited a variant profile consistent with early passages, characterized by a predominance of low-frequency variants in the S gene. In contrast, P53 VAC showed a higher proportion of intermediate- and high-frequency variants compared to earlier passages and included lineage-specific mutations. Some of these variants were not retained in subsequent passages of the main lineage, indicating that alternative patterns of variant emergence and persistence can occur during serial passage.

### 3.5. Dynamics of Variants in the S Gene

Variation in the S gene showed a delayed pattern relative to other genomic regions. Most variants in the S gene emerged during intermediate and late passages and frequently exhibited transient frequency trajectories.

A subset of non-synonymous SNPs in the S gene (9 out of 28) increased in frequency after P60 and remained detectable through P79. Variants were identified in both the S1 and S2 domains, without a clear concentration within the previously defined hypervariable regions of S1.

## 4. Discussion

Serial passage in embryonated eggs remains a widely used strategy for generating live attenuated IBV vaccines, yet the evolutionary processes underlying attenuation and immunogenicity are still incompletely understood [21,22,23,24,25,26]. Attenuation in IBV is widely recognized as a multigenic, strain-specific process. Across IBV lineages, studies consistently show that different combinations of mutations contribute to reduced virulence, with limited overlap among strains [22,27,28].

In this study, we combined in vivo phenotypic characterization with genome-wide, time-resolved analysis of variant dynamics to investigate how viral populations evolve during serial passage and how these changes relate to pathogenicity and immunogenicity.

Our results show that attenuation is associated with progressive changes in variant composition, rather than with a single defining mutation or genomic region. The phenotypic characterization of P7 VIR, P53 VAC, and P79 supports this continuum: P7 VIR retained a virulent phenotype, P53 VAC exhibited attenuation with preserved immunogenicity, and P79 showed a lack of a detectable antibody response, consistent with over-attenuation. Distinct patterns of genomic variation paralleled these phenotypic differences.

A key finding of this study is that viral evolution during serial passage follows a consistent temporal pattern, characterized by sequential phases of variant emergence, expansion, and decline. Early passages were dominated by low-frequency variants, with only a limited number increasing in frequency. These early high-frequency variants were restricted to a small set of loci, primarily in replicase genes and structural genes such as M and 3c (E). This pattern is compatible with early genomic changes associated with replication in the embryonic environment and aligns with previous studies showing that replicase genes play an important role in IBV pathogenicity and host-associated viral dynamics [9,10,11,29].

During intermediate passages, the number of detectable variants increased, reflecting an expansion of the viral mutant spectrum. Multiple variants coexisted within the population, and additional non-synonymous mutations emerged across several replicase components. This phase may represent a period when the mutation supply exceeds the strength of selective filtering, allowing a broader diversification of the viral population [30].

In contrast, late passages showed fewer detectable variants, primarily because low-frequency variants were lost, while previously established variants persisted or reached fixation. This pattern suggests a process of progressive filtering, in which a subset of variants is retained over time while others are eliminated. These dynamics are consistent with the combined effects of selective and stochastic processes acting on viral populations organized as quasispecies.

Although the S protein is a major determinant of host interaction and antigenicity, our results indicate that variation in the S gene accumulated later than in other genomic regions. Most S gene variants emerged during intermediate and late passages and often showed transient frequency trajectories. Only a subset of non-synonymous SNPs increased in frequency in late passages, particularly after P60. Importantly, these changes were not confined to previously defined hypervariable regions of S1, suggesting that Spike evolution during serial passage is not limited to canonical antigenic regions.

The delayed accumulation of Spike variants supports a model in which early changes in the replicase and structural genes establish a genomic background that may influence subsequent variant patterns. Within this framework, variation in the S gene appears to occur on top of a previously established variant composition rather than representing the earliest major source of genomic change during serial passage.

The comparison of derivative lineages further highlights the importance of timing in evolutionary outcomes. P7 VIR, which diverged early, retained a virulent phenotype and lacked late-emerging variants in the S gene, indicating that early mutations alone are insufficient to confer attenuation. In contrast, P53 VAC diverged after the accumulation of early variants but before extensive late-stage genomic changes, consistent with its attenuated yet immunogenic phenotype. These observations suggest that attenuation depends on the accumulation and persistence of mutations over time, rather than solely on the presence of individual mutations.

Notably, P53 VAC and the main lineage at comparable passages did not share identical variant profiles, and some lineage-specific variants were not retained in later passages. This indicates that stochastic processes contribute to evolutionary divergence, even under similar experimental conditions. Such divergence is expected in viral populations that undergo repeated bottlenecks and expansions, where both selection and drift shape variant trajectories.

In addition to point mutations, we identified indels and nonsense substitutions, mostly at a lower frequency. The increasing frequency of the deletion in the 3c (E) gene is consistent with previous reports linking changes in accessory genes to attenuation [31]. In contrast, the Spike truncation previously reported [32] was observed in early passages but did not persist, illustrating that not all mutations that reach detectable frequencies are retained over time and that their fate depends on the accompanying variant composition.

Importantly, the reduced immunogenicity observed in P79 is most likely due to reduced in vivo replication rather than to specific antigenic changes in Spike. Therefore, the relationship between Spike variation and immunogenicity in this system should be interpreted cautiously.

Overall, our results are consistent with a multi-step, genome-wide evolutionary process underlying attenuation, characterized by early accumulation of variants in replication-associated genes, expansion of genomic diversity, and subsequent filtering of variants over time. Within this framework, attenuation reflects the cumulative effects of mutations across multiple genomic regions, shaped by both selective and stochastic processes [33].

From an applied perspective, this genome-wide view of viral evolution provides a framework for identifying passages that balance attenuation and immunogenicity. Monitoring variant dynamics during serial passage may help identify passages with favorable attenuation profiles while reducing the risk of over-attenuation, thereby contributing to more rational development of live attenuated vaccines.

## 5. Conclusions

This study provides a genome-wide view of IBV evolution during serial passage in embryonated chicken eggs. By integrating deep sequencing with phenotypic characterization in SPF chickens, we show that attenuation is associated with progressive shifts in variant composition across the viral genome rather than with a single defining mutation. Our results indicate that serial passage follows reproducible temporal patterns of variant emergence, diversification, and persistence, with delayed accumulation of variations in the S gene relative to other genomic regions. A comparison of virulent, attenuated, and over-attenuated passages further suggests that attenuation and immunogenicity are influenced by the temporal dynamics and persistence of specific variants during passage. Overall, these findings improve our understanding of the evolutionary processes underlying IBV attenuation and support the use of genome-wide variant monitoring to guide the development of live attenuated vaccines.

## Data Availability

Data are available from the corresponding authors upon reasonable request.

## Supplementary Materials

The following supporting information can be downloaded at: https://www.mdpi.com/article/10.3390/vaccines14060467/s1, Table S1: Complete set of variants detected during serial passaging of IBV. All variants identified across the analyzed passages are included, regardless of frequency. For each variant, the gene or coding region, genomic position, nucleotide and/or amino acid change, mutation type, and variant allele frequency (VAF) at each passage are reported.

## References

[1] Beach J.R., Schalm O.W. (1936). A Filterable Virus, Distinct from That of Laryngotracheitis, the Cause of a Respiratory Disease of Chicks. Poult. Sci..

[2] Schalk A. (1931). An Apparently New Respiratory Disease of Baby Chicks. J. Am. Vet. Med. Assoc..

[3] Cavanagh D. (2007). Coronavirus Avian Infectious Bronchitis Virus. Vet. Res..

[4] Cook J.K.A., Jackwood M., Jones R.C. (2012). The Long View: 40 Years of Infectious Bronchitis Research. Avian Pathol..

[5] Valastro V., Holmes E.C., Britton P., Fusaro A., Jackwood M.W., Cattoli G., Monne I. (2016). S1 Gene-Based Phylogeny of Infectious Bronchitis Virus: An Attempt to Harmonize Virus Classification. Infect. Genet. Evol..

[6] Bijlenga G., Cook J.K.A., Gelb J., Wit J.J.D. (2004). Development and Use of the H Strain of Avian Infectious Bronchitis Virus from the Netherlands as a Vaccine: A Review. Avian Pathol..

[7] Britton P., Armesto M., Cavanagh D., Keep S. (2012). Modification of the Avian Coronavirus Infectious Bronchitis Virus for Vaccine Development. Bioengineered.

[8] Casais R., Dove B., Cavanagh D., Britton P. (2003). Recombinant Avian Infectious Bronchitis Virus Expressing a Heterologous Spike Gene Demonstrates That the Spike Protein Is a Determinant of Cell Tropism. J. Virol..

[9] Armesto M., Cavanagh D., Britton P. (2009). The Replicase Gene of Avian Coronavirus Infectious Bronchitis Virus Is a Determinant of Pathogenicity. PLoS ONE.

[10] Hodgson T., Casais R., Dove B., Britton P., Cavanagh D. (2004). Recombinant Infectious Bronchitis Coronavirus Beaudette with the Spike Protein Gene of the Pathogenic M41 Strain Remains Attenuated but Induces Protective Immunity. J. Virol..

[11] Zhao J., Zhang K., Cheng J., Jia W., Zhao Y., Zhang G. (2020). Replicase 1a Gene Plays a Critical Role in Pathogenesis of Avian Coronavirus Infectious Bronchitis Virus. Virology.

[12] Van Beurden S.J., Berends A.J., Krämer-Kühl A., Spekreijse D., Chenard G., Philipp H.-C., Mundt E., Rottier P.J.M., Verheije M.H. (2018). Recombinant Live Attenuated Avian Coronavirus Vaccines with Deletions in the Accessory Genes 3ab and/or 5ab Protect against Infectious Bronchitis in Chickens. Vaccine.

[13] Zhao Y., Cheng J., Yan S., Jia W., Zhang K., Zhang G. (2019). S Gene and 5a Accessory Gene Are Responsible for the Attenuation of Virulent Infectious Bronchitis Coronavirus. Virology.

[14] McKinley E.T., Hilt D.A., Jackwood M.W. (2008). Avian Coronavirus Infectious Bronchitis Attenuated Live Vaccines Undergo Selection of Subpopulations and Mutations Following Vaccination. Vaccine.

[15] Van Santen V.L., Toro H. (2008). Rapid Selection in Chickens of Subpopulations within ArkDPI-Derived Infectious Bronchitis Virus Vaccines. Avian Pathol..

[16] Ghetas A.M., Thaxton G.E., Breedlove C., Van Santen V.L., Toro H. (2015). Effects of Adaptation of Infectious Bronchitis Virus Arkansas Attenuated Vaccine to Embryonic Kidney Cells. Avian Dis..

[17] Reed L.J., Muench H. (1938). A simple method of estimating fifty per cent endpoints. Am. J. Epidemiol..

[18] Callison S.A., Hilt D.A., Boynton T.O., Sample B.F., Robison R., Swayne D.E., Jackwood M.W. (2006). Development and Evaluation of a Real-Time Taqman RT-PCR Assay for the Detection of Infectious Bronchitis Virus from Infected Chickens. J. Virol. Methods.

[19] Li H. (2018). Minimap2: Pairwise Alignment for Nucleotide Sequences. Bioinformatics.

[20] Kearse M., Moir R., Wilson A., Stones-Havas S., Cheung M., Sturrock S., Buxton S., Cooper A., Markowitz S., Duran C. (2012). Geneious Basic: An Integrated and Extendable Desktop Software Platform for the Organization and Analysis of Sequence Data. Bioinformatics.

[21] Ammayappan A., Upadhyay C., Gelb J., Vakharia V.N. (2009). Identification of Sequence Changes Responsible for the Attenuation of Avian Infectious Bronchitis Virus Strain Arkansas DPI. Arch. Virol..

[22] Feng K., Xue Y., Wang J., Chen W., Chen F., Bi Y., Xie Q. (2015). Development and Efficacy of a Novel Live-Attenuated QX-like Nephropathogenic Infectious Bronchitis Virus Vaccine in China. Vaccine.

[23] Geerligs H.J., Boelm G.-J., Meinders C.A.M., Stuurman B.G.E., Symons J., Tarres-Call J., Bru T., Vila R., Mombarg M., Karaca K. (2011). Efficacy and Safety of an Attenuated Live QX-like Infectious Bronchitis Virus Strain as a Vaccine for Chickens. Avian Pathol..

[24] Liu S., Zhang X., Gong L., Yan B., Li C., Han Z., Shao Y., Li H., Kong X. (2009). Altered Pathogenicity, Immunogenicity, Tissue Tropism and 3′-7kb Region Sequence of an Avian Infectious Bronchitis Coronavirus Strain after Serial Passage in Embryos. Vaccine.

[25] Phillips J.E., Jackwood M.W., McKinley E.T., Thor S.W., Hilt D.A., Acevedol N.D., Williams S.M., Kissinger J.C., Paterson A.H., Robertson J.S. (2012). Changes in Nonstructural Protein 3 Are Associated with Attenuation in Avian Coronavirus Infectious Bronchitis Virus. Virus Genes.

[26] Huang Y.P., Wang C.H. (2006). Development of Attenuated Vaccines from Taiwanese Infectious Bronchitis Virus Strains. Vaccine.

[27] Listorti V., Laconi A., Catelli E., Cecchinato M., Lupini C., Naylor C.J. (2017). Identification of IBV QX Vaccine Markers: Should Vaccine Acceptance by Authorities Require Similar Identifications for All Live IBV Vaccines?. Vaccine.

[28] Wang M., Bo Z., Zhang C., Guo M., Wu Y., Zhang X. (2024). Deciphering the Genetic Variation: A Comparative Analysis of Parental and Attenuated Strains of the QXL87 Vaccine for Infectious Bronchitis. Animals.

[29] Keep S., Stevenson-Leggett P., Dowgier G., Everest H., Freimanis G., Oade M., Hammond J.A., Armesto M., Vila R., Bru T. (2022). Identification of Amino Acids within Nonstructural Proteins 10 and 14 of the Avian Coronavirus Infectious Bronchitis Virus That Result in Attenuation In Vivo and In Ovo. J. Virol..

[30] Andino R., Domingo E. (2015). Viral Quasispecies. Virology.

[31] Li H., Qin Y., Han Z., Liu S. (2025). Attenuation and Altered Replication of IBV Strain Tl/CH/LDT3/03 after Serial Passage in Chicken Embryo Fibroblasts and Vero Cells. Virus Res..

[32] Liang R., Liu K., Li Y., Zhang X., Duan L., Huang M., Sun L., Yuan F., Zhao J., Zhao Y. (2024). Adaptive Truncation of the S Gene in IBV during Chicken Embryo Passaging Plays a Crucial Role in Its Attenuation. PLoS Pathog..

[33] Oade M.S., Keep S., Freimanis G.L., Orton R.J., Britton P., Hammond J.A., Bickerton E. (2019). Attenuation of Infectious Bronchitis Virus in Eggs Results in Different Patterns of Genomic Variation across Multiple Replicates. J. Virol..

